# Isoorientin Ameliorates APAP-Induced Hepatotoxicity via Activation Nrf2 Antioxidative Pathway: The Involvement of AMPK/Akt/GSK3β

**DOI:** 10.3389/fphar.2018.01334

**Published:** 2018-11-28

**Authors:** Xiaoye Fan, Hongming Lv, Lidong Wang, Xuming Deng, Xinxin Ci

**Affiliations:** ^1^Institute of Translational Medicine, The First Hospital of Jilin University, Changchun, China; ^2^Key Laboratory of Zoonosis, Ministry of Education, Institute of Zoonosis, College of Veterinary Medicine, Jilin University, Changchun, China

**Keywords:** acetaminophen APAP, hepatotoxicity, Isoorientin Iso, Nrf2, oxidative stress

## Abstract

Oxidative stress has been highlighted as therapeutic targets for acetaminophen (APAP)-induced hepatotoxicity. Isoorientin (Iso), a well-known flavonoid-like compound, has been shown to have antioxidant potential. However, the effect of Iso on APAP-induced liver injury has not yet been elucidated. The present study investigated the hepatoprotective effect of Iso and its underlying mechanism. C57BL/6J mice were used to evaluate the hepatoprotective effect of Iso *in vivo* and HepG2 cells were utilized to further decipher the mechanisms of Iso -induced Nrf2 activation. We found that Iso treatment significantly reduced APAP-induced hepatotoxicity by reducing the lethality, histopathological liver changes, and alanine transaminase (ALT) and aspartate aminotransferase (AST) levels in serum. These effects were accompanied by decreased malondialdehyde (MDA) formation and myeloperoxidase level (MPO), and by decreased superoxide dismutase (SOD) and glutathione (GSH) depletion. Moreover, Iso induced Nrf2 activation and translocation as well as upstream AMPK/Akt/GSK3β activation. Furthermore, Iso effectively alleviated mitochondrial dysfunction by reducing c-jun N-terminal kinase phosphorylation and translocation, Bax mitochondrial translocation, and apoptosis-inducing factor and cytochrome c release. Further mechanistic investigations revealed that the activation of Nrf2 by Iso via the AMPK/Akt/GSK3β pathway contributed to the hepatoprotective activity of Iso *in vitro*. In addition, the Iso-mediated inhibition of APAP-induced the lethality, histopathological changes and mitochondrial dysfunction observed in WT mice was nearly absent in Nrf2^-/-^ mice. In summary, Iso ameliorated APAP-induced hepatotoxicity by activating Nrf2 via the AMPK/Akt/GSK3β pathway.

## Introduction

**FIGURE 1:**
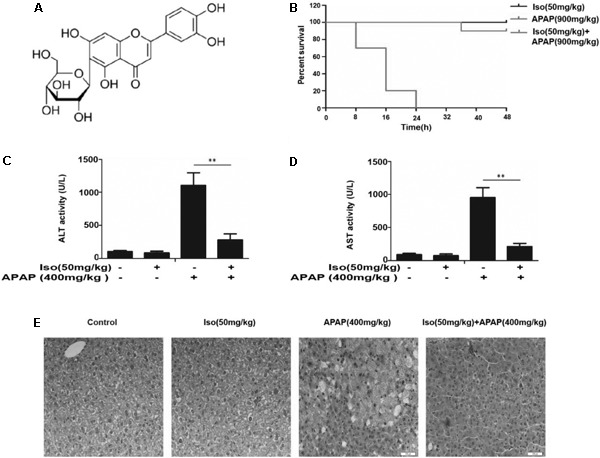
Iso protected against APAP-induced fulminant hepatic failure. **(A)** Chemical structure of Iso. **(B)** Mice were treated with Iso (50 mg/kg i.p.) twice at 12 h intervals. The survival rate of mice was determined by treatment with APAP (900 mg/kg) at a lethal dose 1 h after the last dose of Iso (50 mg/kg). The survival rates of the mice were observed within 48 h after APAP treatment. The percentage of surviving mice is shown for each time point. *n* = 10 in each group. **(C,D)** One hour after the last dose of Iso, APAP (400 mg/kg) was administered for 6 h (*n* = 5/group). We harvested serum for analysis of ALT and AST levels. **(E)** Representative liver histological sections were stained with hematoxylin and eosin (H&E) (400 × magnification). The data represent the average of three independent experiments. All data are presented as the means ± SEM (*n* = 5 in each group). ^∗∗^*p* < 0.01 versus the control group.

Isoorientin (3′,4′,5,7-tetrahydroxy-6-C-glucopyranosyl flavone; Iso), a common C-glycosyl flavone in the human diet, and the chemical structure of Iso is shown in Figure 1A. It can be isolated from several plant species, such as *Phyllostachys pubescens, Patrinia spp.*, buckwheat and corn silks (Yuan et al., 2016). It has been shown that Iso not only exerts anti-inflammatory effects by inhibiting lipopolysaccharide-stimulated cyclooxygenase-2(COX-2) and cytokine production, but also exhibits antioxidant potential (Anilkumar et al., 2017). Previously, we found that Iso treatment exhibits a significant hepatoprotective effect against tertiary-butyl hydroperoxide (t-BOOH)-induced oxidative damage in liver cells and further protects against carbon tetrachloride (CCl_4_)-induced oxidative damage in rats (Orhan et al., 2003). Oxidative stress causes injury in cells by generating reactive oxygen species (ROS), and excessive ROS in the liver can cause extensive liver injury (Chan et al., 2006). Based on the significant pathophysiological role of antioxidation/detoxification system dysfunction in the development of liver diseases, antioxidant agents can be an effective strategy for fighting oxidative stress that causes liver damage and diseases (Lv et al., 2018). Therefore, the ability of Iso to act as an antioxidant with anti-inflammatory activity renders this compound a prime candidate to be a hepatoprotective agent.

In response to oxidative stress caused by ROS, cells have developed adaptive, dynamic processes to maintain cellular redox homeostasis and reduce oxidative damage through a series of antioxidant molecules and detoxifying enzymes (Dunaway et al., 2018). The major pathway that responds to reactive species and redox potential is the Nrf2/ARE pathway, which activates phase II detoxification enzymes at the transcriptional level (Ahmed et al., 2017). Nrf2-deficient animals are more susceptible to organ injury induced by toxic stimuli such as acetaminophen (APAP), benzo[a]pyrene, diesel exhaust and other oxidative stresses due to decreased antioxidant protection (Raghunath et al., 2018). APAP overdose is the leading cause of drug-induced acute liver failure in many developed countries (Budnitz et al., 2011). Given the public concern regarding APAP hepatotoxicity, great efforts have been made to understand the mechanisms of the toxic effects. Mitochondrial oxidative stress and mitochondrial dysfunction are considered to be the predominant cellular processes in APAP hepatotoxicity (Jaeschke et al., 2012). Accordingly, inhibition of oxidative stress and mitochondrial dysfunction may play an essential role in attenuating APAP-induced acute liver injury (AILI). Considering the importance of oxidative stress in APAP-induced hepatotoxicity, we speculate that Nrf2 activators might protect against this toxicity.

In recent few decades, several studies have demonstrated the ability of natural products to counteract oxidative stress and exhibit hepatoprotective activity by modulating the Nrf2/ARE pathway (Kumar et al., 2014; Iranshahy et al., 2018). In view of the importance of free radical stress as a major contributor to the toxicity of APAP, we speculated that Iso has the potential to protect the liver against APAP-induced toxicity. In the current study, we evaluated the effects of Iso on Nrf2 activation and translocation, explored the role of Nrf2 activation in the expression of major antioxidant enzymes, and identified a novel signaling pathway for the regulation of Nrf2 activation. The results of this study using Nrf2 knockout (KO) animal and cell models clearly demonstrate that the ability of Iso to protect hepatocytes from APAP toxicity is dependent on Nrf2 activation.

## Materials and Methods

### Reagents and Chemical

Isoorientin, purity > 98%) was obtained from the Chengdu Pufei De Biotech Co., Ltd. 3-(4,5-Dimethylthiazol-2-y1)-2,5-diphenyltetrazolium bromide (MTT), dimethylsulfoxide (DMSO), Ly294002 (Akt inhibitor) and DCFH-DA were purchased from the Sigma Chemical Co. (St. Louis, Mo, United States). FITC Annexin V Apoptosis Detection Kit was purchased from BD Biosciences. (San Jose, CA, United States).

Antibodies directed against Nrf2 and HO-1 were purchased from Abcam (Cambridge, MA, United States). Anti-phospho-c-Jun NH2-terminal kinase (JNK) antibody and β-actin were obtained from SUNGENE BIOTECH (Tianjin, China). Antibodies against Cytochrome c, Bax, Bcl-2, AMPK, phosphor-AMPK, AKT, phosphor-AKT, GSK-3β and phosphor- GSK-3β were purchased from Cell Signaling (Boston, MA, United States). The horseradish peroxidase (HRP)-conjugated anti-rabbit and anti-mouse IgG were purchased from proteintech (Boston, MA, United States). Additionally, ALT, AST, MDA, MPO, GSH, and SOD test kits were supplied by Nanjing Jiancheng Bioengineering Institute (Nanjing, China).

### Animals and Ethics Statement

Wild-type and Nrf2^-/-^ (knockout) C57BL/6 mice were purchased from Liaoning Changsheng Technology Industrial, Co., Ltd. (Certificate SCXK2010-0001; Liaoning, China) and The Jackson Laboratory (Bar Harbor, ME, United States), respectively. All animals were housed in Specific pathogen free-facility. All animal experiments were carried out in strict accordance with the recommendations in the *Guide for the Care and Use of Laboratory Animals*, which was published by the *United States National Institute of Health*. This study was reviewed and approved by the *Animal Welfare and Research Ethics Committee at Jilin University*.

### Experimental Design and Animal Procedures

Mice were randomly separated into the following four groups (*n* = 10/group): control (saline); Iso (50 mg/kg); APAP only (900 mg/kg or 400 mg/kg); and APAP (900 mg/kg or 400 mg/kg) + Iso (50 mg/kg). The mice were administered Iso (50 mg/kg) i.p. two times for 12 h each time. Then, 1 h after the last dose of Iso, they were exposed to a lethal dose of APAP (900 mg/kg) to observe the mortality rate or treated with APAP (400 mg/kg) for 6 h to evaluate pathological change and biochemical analysis.

### Isolation and Culture of Primary Mouse Hepatocytes

C57BL/6 mice were used in this study. This study was reviewed and approved by the Animal Welfare and Research Ethics Committee at Jilin University. Surgical procedures were conducted under isoflurane-induced anesthesia in an isolated system. Isolated mouse hepatocytes were obtained using a modified collagenase perfusion method (Arbo et al., 2016). Briefly, a cannula was inserted in the hepatic portal vein and the liver was perfused initially with an EGTA-buffer at 37°C, at a constant flow of 5 mL/min. Subsequently, perfusion was continued with a solution of collagenase supplemented by its co-factor calcium until complete digestion. The liver capsule was then gently disrupted to release isolated liver cells into a suspension buffer. The liver cell suspension was subsequently filtered through 100-μm cell strainer and purified through three cycles of low-speed centrifugations (300 rpm for 3 min at 4°C). Cell viability was estimated by the trypan blue exclusion test and was always higher than 80%. A suspension of 0.5 × 10^6^ viable cells/mL was subsequently seeded in 6- or 96-well plates previously coated with collagen (40 μg/mL), in complete culture medium. Cells were incubated overnight at 37°C, with 5% CO_2_, to allow cell adhesion before drug exposure.

### Histopathology Assessment

After bleeding, a portion of fresh liver tissues were fixed in Neutral-buffered formalin, embedded in paraffin and cut into 3 μm sections. The tissue sections were subsequently stained with hematoxylin and eosin (H & E) for microscopic examination.

### Biochemical Assay

The serum/cell supernatant ALT and AST concentrations were measured using an assay kit according to the manufacturer’s instructions. Mouse liver tissues GSH, MPO, MDA and SOD levels according to the manufacturer’s instructions. All results were normalized by the total protein concentration in each sample.

### Cell Culture and MTT Analysis

A hepatoma-derived HepG2 cell line, bought from the China Cell Line Bank (Beijing, China), was cultured in DMEM medium supplemented with 10% FBS, 100 U/mL of penicillin, 100 U/mL of streptomycin and 3 mM glutamine at 37°C in a humidified atmosphere containing 5% CO_2_. Moreover, HepG2 cells (1 × 10^4^ cells/well) were treated with Iso (5, 10, and 20 μM) for 1 h, and exposed to APAP (15 mM) for 20 h. After treatment, cells were incubated with MTT (5 mg/mL) for additional 4 h. Then, the supernatant was removed and the formed blue formazan was dissolved in DMSO. The optical density was measured at 570 nm and Cell viability was normalized as the percentage of control.

### CRISPR/Cas9 Knockout

HepG2 cells were grown in 24-well plates for 16 h, then transfected with a plasmid expressing Cas9 with Nrf2 sgRNA or AMPK sgRNA and a plasmid carrying a puromycin resistant gene using Viafect transfection reagent (Promega). After 36 h, 2 mg/ml puromycin was added to selected cells. Two days later, cells were seeded in 96-well plates at a density of 1 cell per well. Gene editing efficiency after clonal expansion was determined by immunoblotting. DNA sequencing was employed to verify that gene editing was successful.

### Intracellular ROS Measurement

HepG2 cells were seeded into 96-well plates (1 × 10^4^ cells/well) for 24 h, and recovered in serum-free DMEM for 6 h. Next, the cells were subjected to different dosages of Iso (5, 10, or 20 μM) for 18 h, and APAP (15 mM) was added to each well for 3 h. Then the cells were incubated with 50 mM of DCFH-DA for 40 min and DCF fluorescence intensities were assessed by a multi-detection reader at excitation and emission wavelengths of 488 and 535 nm, respectively.

### Quantification of Apoptotic and Necrosis Cells

HepG2 cells were seeded into 12-well plates (5 × 10^5^ cells/well) for 24 h incubation, and then were treated with Iso (5, 10, or 20 μM) 1 h prior to APAP treatment. After 18h, cells were washed twice with ice-cold PBS, and subjected to Annexin V and propidium iodide staining. The percentage of apoptosis and necrosis were determined using flow cytometry (LSR II Flow Cytometer; BD Biosciences, SanJose, CA, United States).

### Western Blot Analysis

The total protein samples from liver tissues were homogenized using RIPA lysis buffer containing protease and phosphatase inhibitors. Cells were plated into 6-well plates and treatment with different concentrations of Iso for the indicated durations. Nuclear and cytoplasmic fractions of cells and liver tissues were obtained using an NE-PER Nuclear and Cytoplasmic Extraction Reagent Kit (Pierce Biotechnology, Rockford, IL, United States) according to the manufacturer’s instructions. Mitochondria Isolation Kit (Sigma-Aldrich, United States) according to the manufacturer’s instructions. A BCA protein assay kit (Beyotime, China) was employed to measure protein concentrations. Equal amounts of proteins were separated by 10% SDS-poly-acrylamide gel, electrophoretically transferred to PVDF membranes and blocked with 5% BSA. Then, the membranes were washed and probed with corresponding primary antibodies and subsequently by secondary antibodies. Protein bands were visualized by ECL. The gray densities of protein bands were normalized by employed β-actin density as an internal control.

### Statistical Analysis

All data referenced above were expressed as the means ± SEM and analyzed using SPSS19.0 (IBM), and One-way analysis of variance (ANOVA) was employed for comparisons between the experimental groups. Statistical significance was defined as *p*^∗^ < 0.05 or *p*^∗∗^ < 0.01.

## Results

### Iso Alleviated APAP-Induced Acute Liver Failure (ALF) *in vivo*

As shown in Figure 1B, the survival rate of the APAP (900 mg/kg) plus Iso (50 mg/kg)-treated mice were much higher than that of the APAP (900 mg/kg)-treated mice. Furthermore, the serum ALT and AST levels were significantly increased in APAP (400 mg/kg)-treated mice, whereas Iso (50 mg/kg) reduced these increases (Figures 1C,D). Histopathological evaluation of the liver further demonstrated the protective effects of Iso against APAP-induced liver damage, including the amelioration of hepatocyte necrosis and hemorrhage (Figure 1E).

### Iso Ameliorated APAP-Induced Oxidative Stress Injury and Induced Nrf2 Transcriptional Activation *in vivo*

**FIGURE 2:**
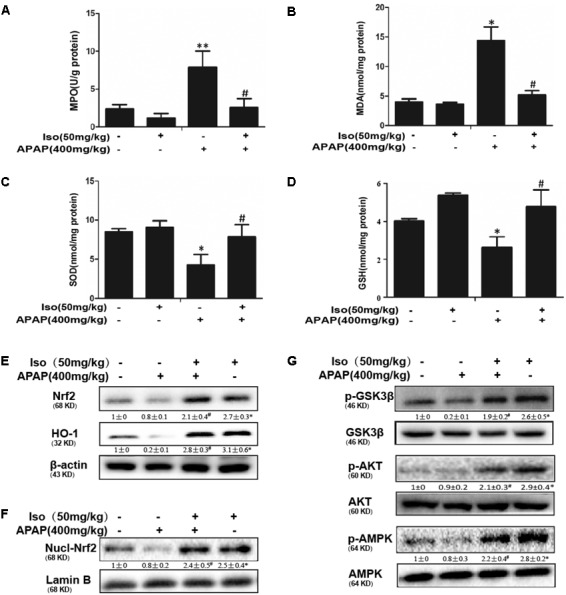
Effect of Iso treatment on the levels of APAP-mediated oxidative stress, Nrf2 activation, and AMPK, Akt and GSK3β phosphorylation in mice. Mice were treated with Iso (50 mg/kg i.p.) twice. One hour after the last dose of Iso, mice received APAP (400 mg/kg). Liver tissues were obtained from the mice 6 h after the APAP challenge for the measurement of MPO and MDA formation and SOD and GSH activity. Furthermore, protein samples were extracted from liver tissue homogenates and analyzed by Western blotting. **(A,B)** Effects of Iso on APAP-mediated MPO and MDA content in liver. **(C,D)** Effects of Iso on APAP-mediated SOD and GSH content in liver. **(E,F)** Effects of Iso on APAP-induced expression and nuclear translocation of Nrf2 and HO-1 protein expression in liver. **(G)** Effects of Iso on APAP-induced AMPK, Akt and GSK-3β phosphorylation in liver. The data represent the average of three independent experiments. Data are presented as the means ± SEM (*n* = 5 in each group). ^∗^*p* < 0.05 and ^∗∗^*p* < 0.01 versus the control group; ^#^*p* < 0.05 and ^##^ *p* < 0.01 versus the APAP group.

As presented in Figure 2, compared to those in the control group, the levels of MDA and MPO were significantly increased in the APAP group, whereas the GSH content and SOD activity were significantly decreased in the APAP group. Our results showed that Iso pretreatment significantly decreased the levels of MPO and MDA (Figures 2A,B) and increased the levels of GSH and SOD (Figures 2C,D), which play vital roles in protecting against APAP-induced oxidative stress. Moreover, APAP challenge mildly suppressed Nrf2 and HO-1 protein expression and Nrf2 translocation, whereas these changes were completely reversed by Iso pretreatment (Figures 2E,F). We further examined the upstream proteins AMPK, AKT and GSK3β, which are involved in the regulation of the Nrf2 activation. As shown in Figure 2G, Iso treatment significantly increased the phosphorylation of AMPK, AKT and GSK3β.

### Iso Ameliorated APAP-Induced Mitochondrial Dysfunction *in vivo*

**FIGURE 3:**
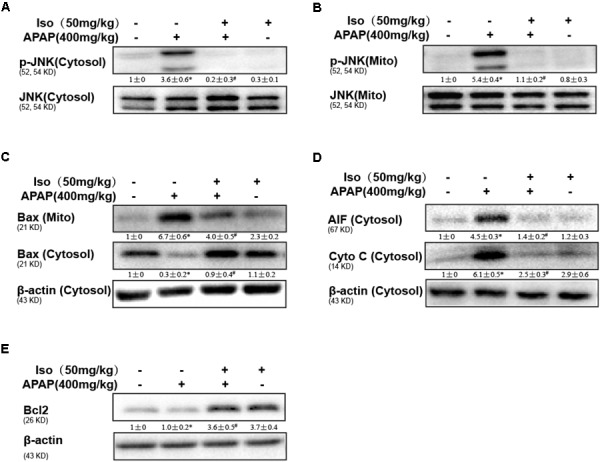
Protective effect of Iso against mitochondrial dysfunction. Mice were treated with Iso (50 mg/kg i.p.) twice. One hour after the last dose of Iso, the mice received APAP (400 mg/kg). **(A,B)** Western blotting was used to detect the levels of phosphorylated JNK in mitochondrial and cytosolic liver fractions at 6 h post-APAP. **(C,D)** Mitochondrial Bax and cytosolic Bax, AIF and cytochrome c were measured by immunoblot analysis at 6 h post-APAP injection. **(E)** Bcl2 in liver tissue lysates was assayed by Western blotting. The results show the average of three independent experiments. ^∗^*p* < 0.05 and ^∗∗^*p* < 0.01 versus the control group; ^#^*p* < 0.05 and ^##^*p* < 0.01 versus the APAP group.

Oxidative stress-induced mitochondrial dysfunction is regarded as a crucial factor in APAP-induced liver injury, so we examined whether Iso pretreatment could inhibit APAP-induced mitochondrial dysfunction. As shown in Figures 3A–D, APAP (500 mg/kg) significantly induced Bax mitochondrial translocation, the release of AIF and cytochrome c, and JNK activation, contributing to mitochondrial dysfunction; however, these effects were effectively inhibited by Iso pretreatment (50 mg/kg). In addition, Bcl-2 was activated by Iso pretreatment, which may be responsible for the hepatoprotective effects of Iso (Figure 3E).

### Effect of Iso on APAP-Induced Cell Viability, Cell Death and Oxidative Stress in HepG2 Cells

**FIGURE 4:**
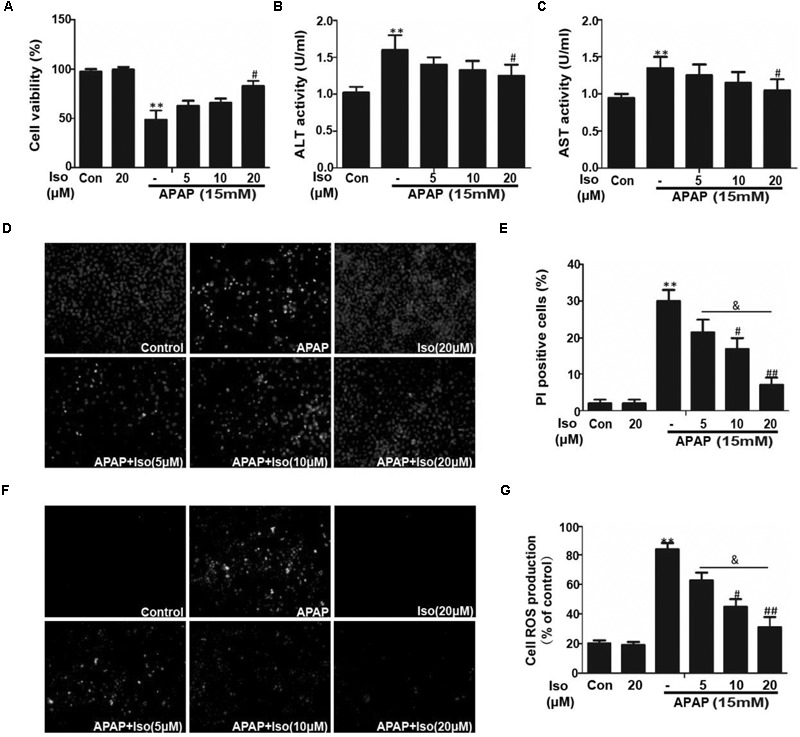
Effect of Iso exposure on APAP-induced cytotoxicity, ALT and AST activities, apoptosis, and oxidative stress in HepG2 cells. **(A)** HepG2 cells were treated with various concentrations of Iso (5, 10, and 20 μM) for 1 h, and the cells received APAP (15 mM) for 24 h. Cell viability was evaluated using an MTT assay. **(B,C)** HepG2 cells were incubated with APAP (15 mM) and various concentrations (5, 10, and 20 μM) of Iso. Cell supernatants were collected after 24 h of incubation to determine ALT and AST activities in the supernatant. **(D,E)** HepG2 cells were treated with various concentrations of Iso (5, 10, and 20 μM) for 1 h, and the cells received APAP (15 mM) for 24 h, and then stained with Hoechst/PI for 15 min. The fluorescence was immediately detected by fluorescence microscope. **(F,G)** HepG2 cells were treated with Iso (5, 10, and 20 μM) for18 h, treated with APAP (15 mM) for 3 h, and then stained with 50 μM DCFH-DA for 40 min. The fluorescence was immediately detected by fluorescence microscope and a multidetection reader. The results show the average of three independent experiments. ^∗∗^*p* < 0.01 versus the control group; ^#^*p* < 0.05 and ^##^*p* < 0.01 versus the APAP group. ^&^*p* < 0.05 versus the Iso (20 μM) plus APAP group.

Cell viability of HepG2 cells co-treated with APAP and Iso were increased compared to control group treated with only APAP. And, when cells were co-treated with Iso, APAP-mediated cytotoxicity was attenuated in a dose-dependent manner (Figure 4A). Primary hepatocytes also have the same result (Supplementary Figure 1C). It is known that ALT and AST are released from damaged hepatocytes. Iso significantly decreased activities of ALT and AST in HepG2 cells due to APAP damage (Figures 4B,C). As shown in Figures 4D,E, APAP (15 mM) caused high cellular mortality and apoptosis, an effect that was inhibited by Iso pretreatment in a dose-dependent manner. Moreover, APAP (15 mM) exposure caused severe oxidative stress, as evidenced by increased ROS generation, whereas Iso markedly suppressed ROS production in a dose-dependent manner (Figures 4F,G).

### Iso Activated Nrf2 and Increased Antioxidant Enzyme Expression *in vitro*

**FIGURE 5:**
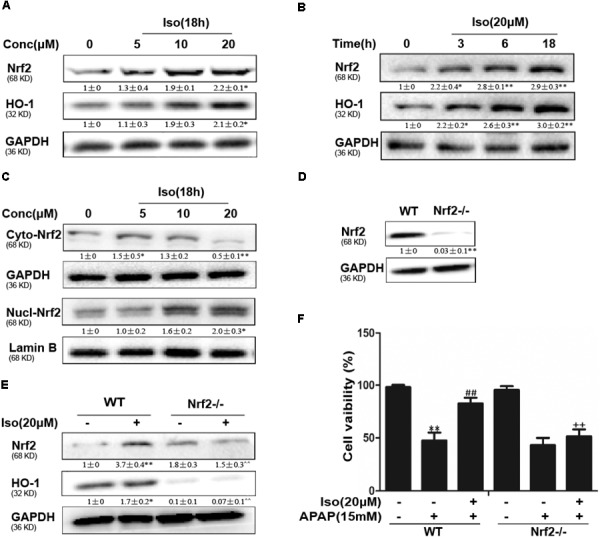
Effect of Iso treatment on Nrf2 expression and nuclear translocation and Nrf2-mediated defense against APAP-stimulated cytotoxicity in HepG2 cells. **(A–C)** HepG2 cells were treated with Iso for the indicated time, and Western blot analysis was used to determine the protein levels. **(D,E)** HepG2 WT and HepG2 Nrf2^-/-^ cells were grown with or without Iso (20 μM) for 18 h; Nrf2 and HO-1 expression was detected by Western blot analysis. **(F)** HepG2 WT and Nrf2^-/-^ cells were treated with Iso (20 μM) for 1 h before APAP (15 mM) treatment for 24 h; cell viability was determined with an MTT assay. All the data represent the average of three independent experiments. ^∗^*p* < 0.05 and ^∗∗^*p* < 0.01 versus the WT control group; ^##^*p* < 0.01 versus the WT APAP group; ^++^*p* < 0.01 versus the WT APAP plus Iso group.

The data in Figures 5A,B show that Iso significantly upregulated Nrf2 and HO-1 protein expression *in vitro* in a dose-dependent manner and in a time-dependent manner, respectively. Primary hepatocytes also have the same result (Supplementary Figure 1A). Moreover, Iso induced Nrf2 translocation in a dose-dependent manner (Figure 5C). An increasing body of evidence indicates that Nrf2 plays a key role in HO-1 regulation. Therefore, a plasmid expressing Cas9 with Nrf2 sgRNA was used to knock out Nrf2 expression in HepG2 cells. The results of Western blot analysis showed that the upregulation of Nrf2 and HO-1 expression induced by Iso was markedly suppressed in Nrf2^-/-^ cells compared to control cells (Figures 5D,E). Furthermore, the protective effect of Iso against APAP-induced cell death in the HepG2 WT cells was abolished in the HepG2 Nrf2^-/-^ cells (Figure 5F).

### Iso Enhanced Cell Viability Through an AMPK/AKT/Nrf2-Dependent Mechanism *in vitro*

**FIGURE 6:**
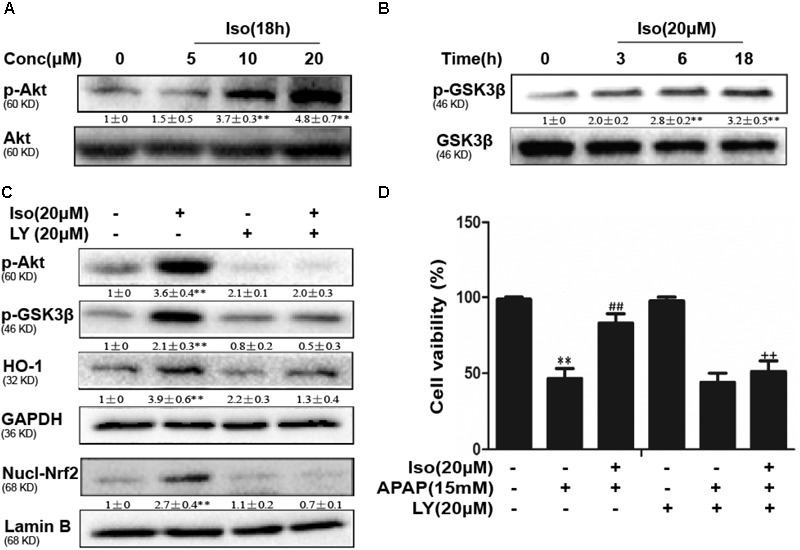
Effect of Iso exposure on AKT-mediated GSK3β phosphorylation and the critical role in Iso-mediated protection against APAP-induced cytotoxicity in HepG2 cells. **(A,B)** HepG2 cells were incubated for 18 h with Iso, and the levels of AKT and GSK3β phosphorylation were determined by Western blot analysis. **(C)** HepG2 cells were pretreated with LY294002 (20 μM) for 18 h and then treated with Iso for 18 h; Western blot analysis was used to determine the levels of AKT and GSK3β and the extent of Nrf2 nuclear translocation. **(D)** The cells were pretreated with LY294002 (20 μM) for 1 h before treatment with Iso. After 1 h, the cells were received APAP for 24 h, and cell viability was measured with an MTT assay. The results show the average of three independent experiments. ^∗∗^*p* < 0.01 versus the control group; ^##^*p* < 0.01 versus the APAP group; ^++^*p* < 0.01 versus the APAP plus Iso group.

**FIGURE 7:**
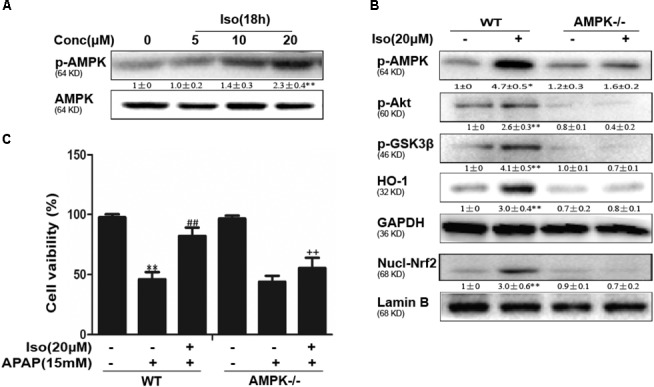
Iso-induced AMPK phosphorylation is essential for AKT/GSK3β-mediated Nrf2 activity and cytoprotection in HepG2 cells. **(A)** HepG2 cells were cultured with Iso for 18 h. Western blot analysis was used to detect the expression of p-AMPK and AMPK. **(B)** HepG2 WT and AMPK^-/-^ cells were incubated with or without Iso (20 μM) for 18 h; Western blot analysis was used to detect the phosphorylation of AMPK, Akt, GSK3β and Nrf2. **(C)** HepG2 WT and AMPK^-/-^ cells were pretreated with 20 μM Iso for 1 h before APAP (15 mM) treatment for 24 h; cell viability was determined with an MTT assay. All the data presented as the average of three independent experiments. ^∗^*p* < 0.05 and ^∗∗^*p* < 0.01 versus the WT control group; ^##^*p* < 0.01 versus the WT APAP group; ^++^*p* < 0.01 versus the WT APAP plus Iso group.

Several mechanisms are involved in the regulation of Nrf2 activation, such as the phosphorylation of Akt. We further examined the effect of Iso on AKT phosphorylation and Akt-mediated GSK3β inhibitory phosphorylation. As shown in Figures 6A,B, Iso significantly increased AKT and GSK3β phosphorylation in a dose-dependent manner. AMPK acts upstream of AKT to increase the inhibitory phosphorylation of GSK-3β and could be activated by Iso in a dose-dependent manner (Figure 7A). Primary hepatocytes also have the same result (Supplementary Figure 1B). In addition, preincubation of cells with LY294002 (an AKT inhibitor) or HepG2 AMPK^-/-^ cells prevented the Iso-induced Nrf2/HO-1 activation and cytoprotection (Figures 6C,D, 7B,C).

### The Suppressive Effects of Iso on APAP-Induced Liver Injury Were Dependent on Nrf2

**FIGURE 8:**
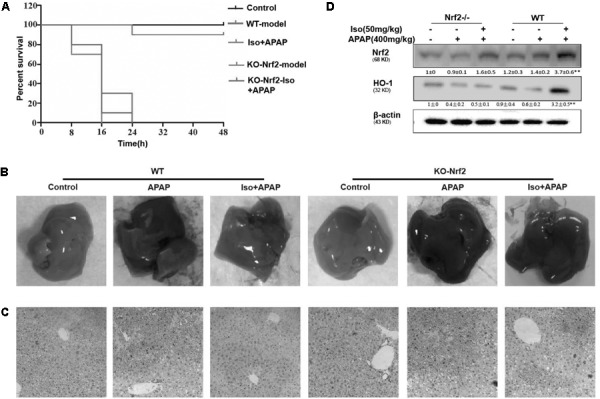
Protective effects of the Iso-mediated regulation of Nrf2 against APAP-induced AILI in mice. WT and Nrf2^-/-^ mice were intraperitoneally injected with Iso (50 mg/kg) twice at 12 h intervals. **(A)** Survival rate of WT and Nrf2^-/-^ mice that received a lethal dose of APAP (900 mg/kg) 1 h after the last dose of Iso (50 mg/kg). The data represent the percentage of surviving mice at each time point. *n* = 15 in each group. **(B)** One hour after the last dose of Iso and 6 h after the APAP (400 mg/kg) challenge, livers (*n* = 5) from each experimental group were processed for gross examination. **(C)** Representative histological sections of the liver were stained with hematoxylin and eosin (H&E). **(D)** Effects of Iso on the protein expression of Nrf2 and HO-1. All data are presented as the means ± SEM (*n* = 5 in each group). ^##^ *p* < 0.01 versus the control group; ^∗∗^*p* < 0.01 versus the APAP group.

The dependency of the hepatoprotective role of Iso on Nrf2 was further assessed in WT and Nrf2^-/-^ mice. First, as shown in Figure 8A, Nrf2^-/-^ mice appeared to be more vulnerable to APAP-induced lethality than WT mice, as Nrf2 knockout reduced the survival rate from approximately 20% to approximately 0. Second, for WT mice, the final survival rates were 20 and 90% for the vehicle group and the Iso-treated group, respectively, while, for Nrf2^-/-^ mice, the final survival rates were 0% in both the vehicle group and the Iso -treated group. We further examined Nrf2 and HO-1 expression and histopathological changes in the liver. As shown in Figures 8B–D, the inhibitory effects of Iso were abolished in Nrf2^-/-^ mice.

## Discussion

Natural products have made enormous contributions to drug discovery, as they have many advantages over conventional chemical compound-based medications, such as fewer side effects, less long-term toxicity, and variable bioavailability and biological activity (Kim, 2018). In recent years, intensive studies have demonstrated the protective effects of natural products against APAP-induced hepatotoxicity due to their multiple mechanisms of action in inflammation, oxidant/antioxidant balance and damage responses (Ko et al., 2017; Lv et al., 2018). Iso (or homoorientin) is a chemical flavonoid-like compound that has been shown to exert anti-inflammatory effects and exhibit antioxidant potential (Ye et al., 2016). To our knowledge, no report has investigated the protective activity of Iso against APAP-induced hepatotoxicity. The present study aimed to investigate the protective effects of Iso against APAP-induced hepatotoxicity and further explore the molecular mechanisms *in vivo* and *in vitro*.

APAP overdose-induced liver dysfunction is the most common cause of drug-induced liver injury (DILI) worldwide, which is characterized by lethality, high ALT and AST levels in serum and pathological changes in the liver (Brodsky et al., 2009). Using an APAP-induced mouse model, we found that APAP markedly increased mortality, serum ALT and AST levels and liver histopathology changes, as reported previously (Zhao et al., 2018). However, Iso treatment significantly prevented these elevations, suggesting that Iso protected liver tissues against the toxic effects of APAP. An abundance of evidence has shown that oxidative stress contributes to histopathological changes by inducing MDA and MPO formation and by decreasing in hepatic levels of SOD and GSH after APAP challenge (Wang L. et al., 2016; Xu et al., 2017). The results of our investigation show that Iso significantly reduced MDA and MPO levels and reversed GSH and SOD depletion, indicating that Iso treatment dramatically alleviated APAP-induced oxidative injury in mice. Oxidative stress-mediated JNK activation and mitochondrial dysfunction play central roles in APAP-induced hepatic injury (Saito et al., 2010); in these processes, JNK phosphorylation enhances the release of cytochrome c and AIF from the mitochondria and enhances Bax translocation to the mitochondria. Western blot analyses in liver tissue clearly demonstrated that Iso suppressed JNK phosphorylation, Bax mitochondrial translocation, and AIF and cytochrome c release. Together, the current results emphasize that Iso may serve as a hepatoprotective agent for inhibiting oxidative stress and mitochondrial dysfunction.

Iso has been reported to possess a notable hepatoprotective effect mediated by the regulation of the respiratory chain complexes and the activity of phase II detoxifying enzymes (Yuan et al., 2016). Nrf2, as a coordinator of multiple signaling pathways, plays a key role in maintaining cellular redox homeostasis and defensing against oxidative stress. Nrf2 activation is observed in hepatic stellate cells, Kupffer cells and parenchymal hepatocytes (Yeligar et al., 2010). We further investigated the critical involvement of Nrf2 in Iso-mediated protection against APAP-induced liver injury. Our data showed that Iso induced Nrf2 and HO-1 activation and translocation in liver tissue. We further determined the upstream processes involved in Nrf2 activation by Iso. Notably, AMPK, a sensor of cellular energy status, regulates cell survival and death in the presence of oxidative stress (Shirwany and Zou, 2014). Iso has been reported to activate AMPK in pancreatic cancer cells (Ye et al., 2016). In our study, Iso treatment significantly increased the phosphorylation of AMPK, AKT, and GSK-3β, which might contribute to Nrf2 activation and hepatoprotective effects *in vivo*. Previous studies have demonstrated that a suite of signaling pathways play important roles in the process of hepatotoxicity, including GSK3β/Nrf2 (Mobasher et al., 2013), PKCzeta-Akt-GSK3beta (Shieh et al., 2009) and AMPK/Akt/GSK3β (Wang L. et al., 2016) pathways. Such studies may provide potential targets for pharmacological candidates for the treatment of liver injury.

To further determine the functional role of Nrf2 in the hepatoprotective effects of Iso and to decipher the mechanisms of Iso-induced Nrf2 activation, we utilized HepG_2_ cells challenged with APAP *in vitro*. As shown in Figure 4, Iso significantly suppressed APAP-induced cytotoxicity, apoptosis and ROS generation. In addition, Iso increased Nrf2 expression and nuclear translocation as well as HO-1 expression in a dose-dependent manner. Considering the key role of Nrf2 in HO-1 regulation and in hepatoprotective effects (Wang W. et al., 2016), we speculated that the upregulation of HO-1 expression and the suppression of cytotoxicity were dependent on Nrf2 activation. This hypothesis was verified by the finding that the Iso-induced cytoprotection and increased HO-1 expression that occurred in normal HepG2 cells were abolished in HepG2 Nrf2^-/-^ cells. Together, these results suggest that Iso protects against cytotoxicity and that oxidative stress depends on Nrf2 activation. Given the results of our *in vivo* study, we further explored the effect of the AMPK/Akt/GSK3β pathway on Iso-induced Nrf2 activation *in vitro*. Our results demonstrated that Iso induced AMPK, Akt, GSK3β, and Nrf2 activation *in vitro*, and the Nrf2 activation and cytoprotective effect of Iso were abolished by an AKT inhibitor or by AMPK knockout (Figures 6, 7). A large number of natural products process potent anti-APAP-induced hepatotoxicity, such as dioscin. The difference is that dioscin showed a remarkable protective effect against APAP-induced hepatotoxicity by adjusting mitochondrial function (Zhao et al., 2012), and Iso ameliorated APAP-induced hepatotoxicity by activating Nrf2 via the AMPK/Akt/GSK3β pathway.

Given all of these results, we used Nrf2-deficient mice to further elucidate whether the inhibition of APAP-induced hepatotoxicity by Iso involves Nrf2 activation. Unlike in WT mice, Iso could not alleviate the mortality or histopathological changes in the liver associated with APAP challenge in Nrf2-deficient mice. In summary, our findings demonstrated the hepatoprotective effect of Iso against APAP-induced oxidative damage and mitochondrial dysfunction, an effect that may be strongly dependent on Iso-mediated Nrf2 activation.

## Author Contributions

XF and HL conducted the experiments and wrote the paper. LW conducted the experiments. XD and XC contributed to the design and improvement of the experiments.

## Conflict of Interest Statement

The authors declare that the research was conducted in the absence of any commercial or financial relationships that could be construed as a potential conflict of interest.
