# Supplementary material for: Isoorientin Ameliorates APAP-Induced Hepatotoxicity via Activation Nrf2 Antioxidative Pathway: The Involvement of AMPK/Akt/GSK3β
Source: Front Pharmacol. 2018 Nov 28;9:1334. doi: 10.3389/fphar.2018.01334 (PMC6279939; doi:10.3389/fphar.2018.01334)
Supplement: Supplementary file 1 [file Data_Sheet_1.PDF]

Isoorientin ameliorates APAP-induced hepatotoxicity via activation Nrf2 antioxidative pathway: the involvement of AMPK/Akt/GSK3β

Xiaoye Fan[a,b,1], Hongming Lv[a,b,1], Lidong Wang[a], Xuming Deng[b], Xinxin Ci[a]*

[a] Institute of Translational Medicine, The First Hospital of Jilin University, Changchun, China.

[b] Key Laboratory of Zoonosis, Ministry of Education, Institute of Zoonosis, College of Veterinary Medicine, Jilin University, Changchun, China

**corresponding author.*

E-mail address: cixinxin@jlu.edu.cn

Full postal address: Dongminzhu road 519, The First Hospital of Jilin University, Changchun, Jilin, 130001, China.

[1] These authors contributed equally to this work.

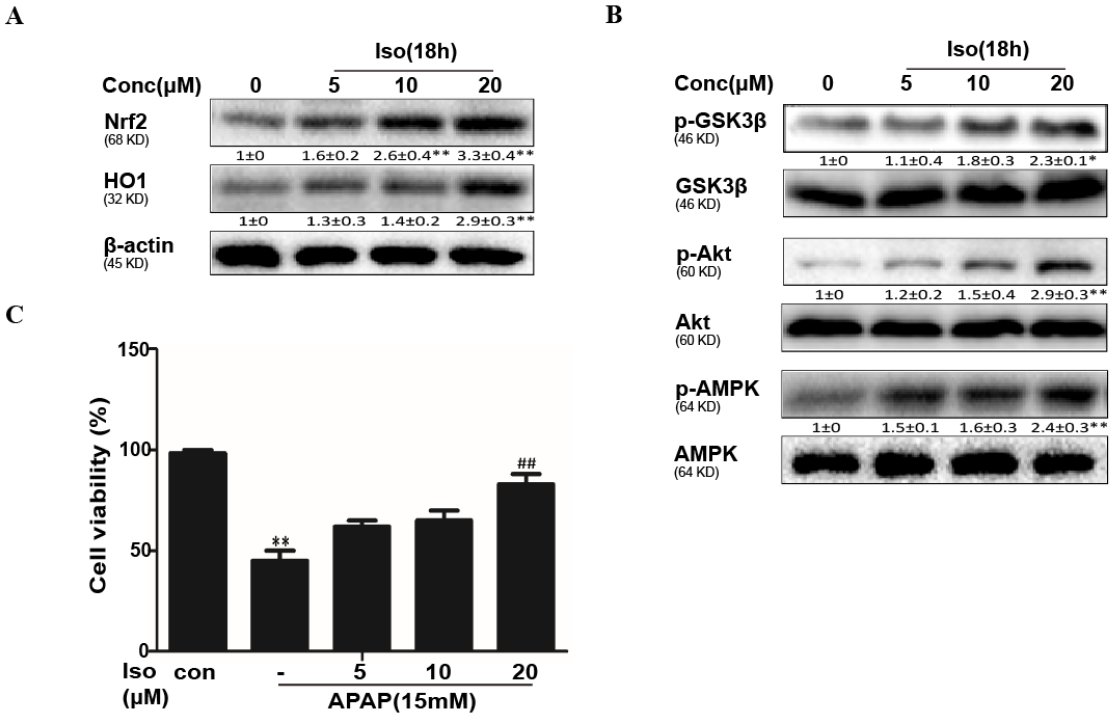

EXTENDED DATA FIGURE 1 | Effect of Iso exposure on Nrf2 activation, and AMPK, Akt and GSK3β phosphorylation, and Iso-mediated protection against APAP-induced cytotoxicity in primary hepatocytes. **(A, B)** Primary hepatocytes were treated

with Iso for the indicated time, and Western blot analysis was used to determine the protein levels. **(C)** Primary hepatocytes were treated with various concentrations of Iso (5, 10 and 20 μM) for 1 h, and the cells received APAP (15 mM) for 24 h. Cell viability was evaluated using an MTT assay. The results show the average of three independent experiments. $^{*}p<0.05$ and $^{**}p<0.01$ versus the control group; $^{\#\#}p<0.01$ versus the APAP group.
